# Non-*g* Factors Predict Educational and Occupational Criteria: More than *g*

**DOI:** 10.3390/jintelligence6030043

**Published:** 2018-09-07

**Authors:** Thomas R. Coyle

**Affiliations:** Department of Psychology, University of Texas at San Antonio, One UTSA Circle, San Antonio, TX 78249, USA; thomas.coyle@utsa.edu; Tel.: +1-210-458-7407; Fax: +1-210-458-5728

**Keywords:** general intelligence (*g*), non-*g* factors, specific abilities, ability tilt, non-*g* residuals

## Abstract

In a prior issue of the *Journal of Intelligence*, I argued that the most important scientific issue in intelligence research was to identify specific abilities with validity beyond *g* (i.e., variance common to mental tests) (Coyle, T.R. Predictive validity of non-*g* residuals of tests: More than *g*. *Journal of Intelligence* 2014, *2*, 21–25.). In this Special Issue, I review my research on specific abilities related to non-*g* factors. The non-*g* factors include specific math and verbal abilities based on standardized tests (SAT, ACT, PSAT, Armed Services Vocational Aptitude Battery). I focus on two non-*g* factors: (a) *non-g residuals*, obtained after removing *g* from tests, and (b) *ability tilt*, defined as within-subject differences between math and verbal scores, yielding math tilt (math > verbal) and verbal tilt (verbal > math). In general, math residuals and tilt positively predict STEM criteria (college majors, jobs, GPAs) and negatively predict humanities criteria, whereas verbal residuals and tilt show the opposite pattern. The paper concludes with suggestions for future research, with a focus on theories of non-*g* factors (e.g., investment theories, Spearman’s Law of Diminishing Returns, Cognitive Differentiation-Integration Effort Model) and a magnification model of non-*g* factors.

## 1. Introduction

This paper begins with the parable of the blind men and an elephant. In the original parable, a group of blind men touch different parts of an elephant and reach different conclusions. One man touches the tusk and believes the elephant is a spear; another touches a leg and believes it is a tree; yet another touches the trunk and believes it is a snake. A modified version of the parable can illustrate a key problem in intelligence research: distinguishing general intelligence (*g*) and specific abilities. In the modified version, the elephant represents *g* and its parts represent specific abilities such as math ability, verbal ability, and spatial ability. The blind men are intelligence researchers who focus on a specific ability, ignoring the overlap between the specific ability and *g*. These “blind” intelligence researchers may incorrectly conclude that the specific ability predicts a criterion when it derives its predictive power entirely from *g*.

A lesson of the modified parable is that the predictive power of a specific ability (beyond *g*) can only be assessed *after removing g*, which is related to all cognitive abilities. The current paper reviews research on the predictive power of specific abilities for diverse criteria (e.g., college grades, college majors, jobs) after removing *g*. The focus is on specific abilities (e.g., math and verbal) measured by standardized tests. The tests include the SAT (formerly, Scholastic Aptitude Test) and ACT (formerly, American College Test), two college admissions tests taken by high school students; the Preliminary SAT (PSAT), an eligibility test used by the National Merit Scholarship Program and taken by high school students; and the Armed Services Vocational Aptitude Battery (ASVAB), a selection test used by the US Armed Forces. The SAT, ACT, PSAT, and ASVAB are strongly related to IQ and *g* and are available in datasets with large and representative samples such as the National Longitudinal Survey of Youth (NLSY) (e.g., [1], p. 19; see also, [2,3]).

The focus on non-*g* factors is consistent with my view that the most important scientific issue in intelligence research is to identify non-*g* factors with validity beyond *g* (cf. [4], p. 21). As discussed below, my research on non-*g* factors calls into question the primacy of *g* hypothesis, which assumes that *g* explains the predictive power of cognitive tests and that non-*g* factors have negligible predictive power (cf. [5]). In contrast to this hypothesis, my research shows that non-*g* factors predict diverse criteria, that non-*g* effects are substantial in size (βs ≈ 0.30), and that non-*g* effects are consistent with theories of intelligence (e.g., investment theories).

The paper is divided into four sections. The first section discusses the predictive validity of *g* and non-*g* factors. The second section reviews a key study [6] that launched my research program on non-*g* factors. The next three sections discuss my subsequent research on non-*g* factors, ending with a review of studies by other researchers. The final section discusses directions for future research, highlighting theories of non-*g* factors and a magnification model of non-*g* factors.

## 2. *g* and Non-*g* Factors: The Primacy of *g*

A key distinction in intelligence research is between *g*, which represents variance common to cognitive tests, and non-*g* factors, which represent variance obtained after (statistically) removing *g* from tests. *g* can be identified in a factor analysis of diverse cognitive tests, which typically shows that the first factor (dubbed *g*) explains more variance among tests than any other factor (e.g., [7], pp. 73–88). The basis of *g* is *positive manifold*. Positive manifold refers to positive correlations among diverse cognitive tests, which indicate that people who do well on one test tend to do well on all others.

*g* is one of the best predictors of school and work performance (for a review, see [7], pp. 270–305; see also, [8,9]). Moreover, a test’s *g* loading (i.e., its correlation with *g*) is directly related to its predictive power. In general, tests with strong *g* loadings correlate strongly with school and work criteria, whereas tests with weak *g* loadings correlate weakly with such criteria. For example, Jensen ([7], p. 280) found that the *g* loadings of the Wechsler Adult Intelligence Scale (WAIS) subtests were directly related to their predictive power for school criteria (e.g., school grades and class ranks). WAIS subtests with stronger *g* loadings generally predicted school criteria well, whereas subtests with weaker *g* loadings predicted such criteria poorly. Consistent with these findings, Thorndike [10] found that *g* explained most of the predictable variance in academic achievement (80–90%), whereas non-*g* factors (obtained after removing *g* from tests) explained a much smaller portion of variance (10–20%). Similar results have been found for job training and productivity, which are robustly related to *g* but negligibly related to non-*g* factors of tests (e.g., *r*_non-*g*_ < 0.10, [7], pp. 283–285; see also, [9,11]).

The totality of evidence supports the primacy of *g* hypothesis, which assumes that *g* largely explains the predictive power of tests and that non-*g* factors have limited or negligible predictive power. Contrary to the primacy of *g* hypothesis, my research shows that non-*g* factors of standardized tests (e.g., SAT, ACT, PSAT) robustly predict educational and occupational criteria, with non-*g* effects often being substantial in size (βs ≈ 0.30).^1^

## 3. A Foundational Study by Coyle and Pillow [6]: Non-*g* Residuals Predict College GPA

Non-*g* factors are operationalized as factors obtained after statistically removing *g* from tests. In the current paper, the focus is on non-*g* factors of standardized tests drawn from the 1997 NLSY (*N* = 8989). The tests include the SAT, ACT, PSAT, and ASVAB. Special attention is given to the SAT and ACT, two college admissions tests that measure math and verbal abilities. The SAT and ACT correlate moderately with college GPA (*r* = 0.43) and strongly with IQ tests and a *g* based on the ASVAB (*r* = 0.78) ([6], p. 274; see also, [2,3]). The ASVAB is a selection test used by the US Armed Forces. It includes 12 diverse cognitive tests, which measure two academic abilities (math and verbal) and two non-academic abilities (shop/technical skills and mental speed). In most studies (described below), non-*g* factors of the SAT, ACT, and PSAT are obtained after removing a *g* based on the ASVAB and are correlated with the specific abilities of the ASVAB and with other criteria (e.g., college majors and jobs).

A foundational study by Coyle and Pillow [6] examined the predictive power of non-*g* residuals of the SAT and ACT (obtained after removing *g*) for first-year college GPA. The study is foundational in the sense that it precipitated my later research, which examined other non-*g* factors and other criteria (e.g., specific GPAs, college majors, jobs). The study has an interesting history. The initial results were obtained using simple regressions and data from a university sample. The analysis regressed college GPA on SAT and ACT scores after removing *g* (*g* was based on the Wonderlic, a word recall test, and other tests). Surprisingly, the SAT and ACT predicted college GPA after removing *g*, which generally explains the predictive power of tests (e.g., [7], pp. 270–305).

**Figure 1:**
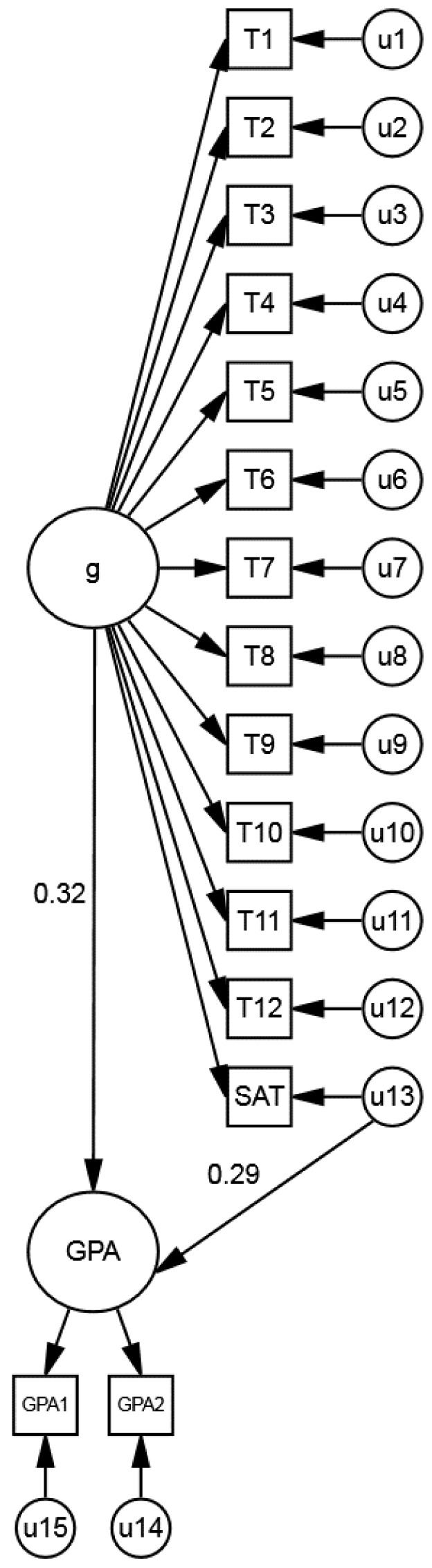
Model of *g* with the SAT, ASVAB tests (T1–T12), and college GPA. A parallel model (not shown) analyzed the ACT. The symbol “u13” represents the non-*g* residuals of SAT composite scores (math + verbal), obtained after removing *g*. The u13→GPA path estimates the relation of the SAT non-*g* residuals with GPA (β = 0.29). Figure adapted from Coyle and Pillow [6].

The results were submitted to *Intelligence* and returned with suggestions for revisions. A key suggestion was to replicate the results with a more representative sample and a more sophisticated analytical approach. The NLSY was identified as a good data source because it contained a large and representative sample (*N* = 8989) as well as college GPAs, SAT and ACT scores, and ASVAB scores. Using the NLSY, structural equation modeling estimated *g* and non-*g* factors. *g* was estimated using the ASVAB, and the non-*g* residuals of the SAT and ACT (obtained after removing *g*) were correlated with college GPA (Figure 1). The key result was that the non-*g* residuals of the SAT and ACT predicted college GPA almost as well as *g* predicted college GPA (βs ≈ 0.30).^2^ The results are inconsistent with the primacy of *g* hypothesis, which assumes that non-*g* factors have negligible predictive power (cf. [5]).

What might explain the predictive power of SAT and ACT non-*g* residuals (for college GPA)? One possibility is that the SAT and ACT measure specific abilities with predictive power for college GPA, which reflects an amalgam of traits. Such traits include math and verbal abilities, which are a staple of college curricula and may predict college GPA. This possibility led to subsequent research (discussed below), which focused on the predictive power of non-*g* residuals of the SAT and ACT math and verbal subtests.

## 4. Non-*g* Residuals of the SAT and ACT Predict Specific Abilities and GPAs

**Figure 2:**
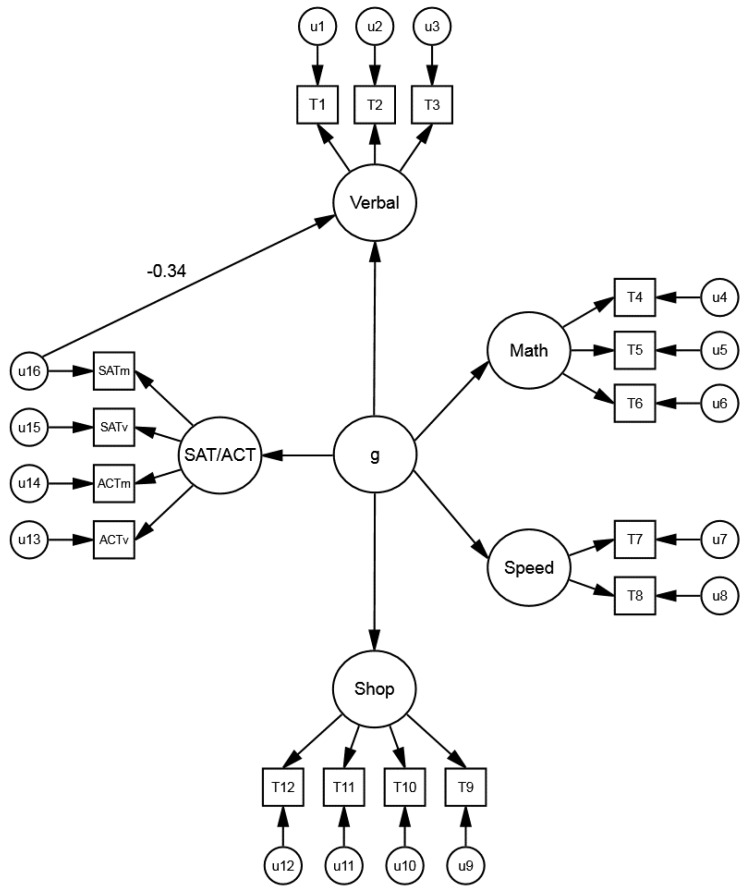
Model of *g* with the SAT subtests, ACT subtests, ASVAB abilities. The symbol “u16” represents the SAT math non-*g* residuals (based on the math subtest), obtained after removing *g*. The u16→Verbal path estimates the relation of the SAT math non-*g* residuals with ASVAB verbal ability (β = −0.34). Figure adapted from Coyle et al. [15].

The study by Coyle and Pillow [6] fueled additional research on non-*g* residuals. In a subsequent study, Coyle, Purcell, Snyder, and Kochunov [15] examined the predictive power of non-*g* residuals of the SAT and ACT math and verbal subtests (obtained after removing *g*) for specific abilities on the ASVAB. The ASVAB consisted of 12 tests: arithmetic reasoning (AR), assembling objects (AO), auto information (AI), coding speed (CS), electronics information (EI), general science (GS), math knowledge (MK), mechanical comprehension (MC), numerical operations (NO), paragraph comprehension (PC), shop information (SI), and word knowledge (WK). These tests estimated four abilities (indicators): verbal ability (GS, PC, WK), math ability (AR, AO, MK), shop ability (AI, EI, SI, MC), and mental speed (CS, NO). The four abilities were correlated with the non-*g* residuals of the SAT and ACT math and verbal subtests (Figure 2).

Coyle et al. [15] found a domain-specific pattern of effects between the non-*g* residuals of the SAT and ACT subtests and the math and verbal abilities of the ASVAB. The math residuals of the SAT and ACT correlated positively with math ability (*M*_β_ = 0.29) and negatively with verbal ability (*M*_β_ = −0.32). In contrast, the verbal residuals of the SAT and ACT correlated positively with verbal ability (*M*_β_ = 0.29) and negatively with math ability (*M*_β_ = −0.25) (The non-*g* residuals of the SAT and ACT correlated negligibly with the ASVAB shop and speed abilities, demonstrating discriminant validity).

Coyle et al. [15] interpreted the results in terms of investment theories ([16], pp. 138–146), which assume that investment in a specific ability (e.g., math) boosts similar abilities but retards competing abilities (e.g., verbal). Math residuals presumably reflect investment in math, which boosts math ability. In contrast, verbal residuals presumably reflect investment in verbal areas, which boosts verbal ability. In addition, because time is limited, investment in one ability (math) comes at the expense of investment in competing abilities (verbal), yielding negative relations between competing abilities (e.g., math and verbal).

**Figure 3:**
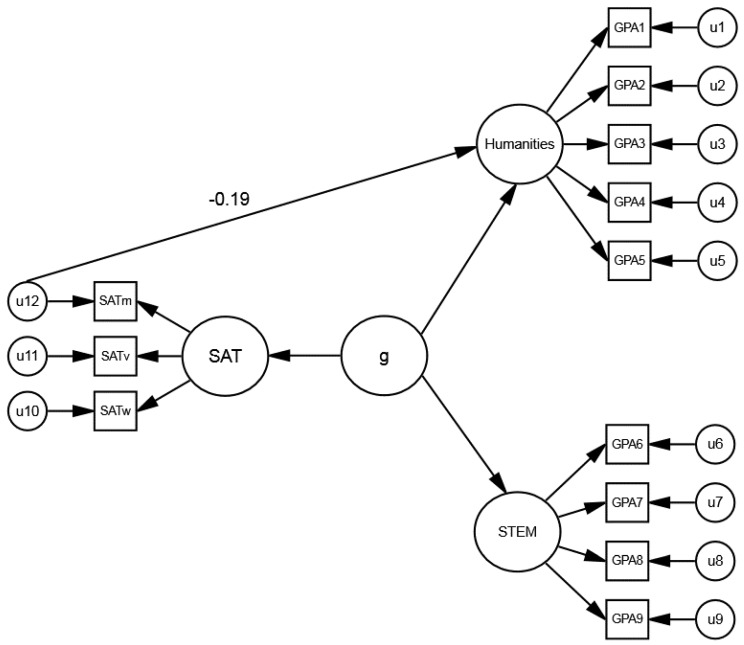
Model of *g* with STEM and humanities GPA factors. *g* was based on an SAT factor, estimated using SAT scores; a STEM factor, estimated using STEM GPAs, and a humanities factor, estimated using humanities GPAs. The non-*g* residuals of the SAT subtests, obtained after removing *g*, were correlated with the STEM and humanities factors. The model shows the relation of the SAT math non-*g* residuals with the humanities factor (β = −0.19). Figure adapted from Coyle, Snyder, Richmond, and Little [17].

Would Coyle et al.’s [15] results be replicated with college grades, which the SAT and ACT were designed to predict? This question was addressed by Coyle, Snyder, Richmond, and Little [17], who examined relations of SAT math and verbal non-*g* residuals with subject specific GPAs, using the College Board Validity Study dataset (*N* = 160,670). SAT scores were obtained for the math, reading, and writing subtests. College GPAs were obtained for courses in two categories: science, technology, engineering, and math (STEM), which were math loaded, and humanities, which were verbally loaded. *g* was based on an SAT factor, estimated using SAT scores; a STEM factor, estimated using STEM GPAs (e.g., math, science, engineering); and a humanities factor, estimated using humanities GPAs (e.g., English, history, foreign languages) (Figure 3). The non-*g* residuals of each SAT subtest (obtained after removing *g*) were correlated with the STEM and humanities factors.

Coyle, Snyder, Richmond, and Little’s [17] results confirmed the domain-specific pattern obtained with the ASVAB abilities. SAT math residuals correlated positively with the math-based STEM GPA factor and negatively with the verbal-based humanities GPA factor. Conversely, SAT verbal residuals (reading and writing) showed the opposite pattern. The mean absolute effect (|*M*_β_| ≈ 0.17) was smaller than the mean absolute effect for the ASVAB abilities (|*M*_β_| ≈ 0.29) (cf. [15]). (The smaller effect could be attributed to the use of GPAs, which are less reliable than standardized test scores.) The results confirm the predictive power of non-*g* residuals and are inconsistent with the primacy of *g* hypothesis, which assumes that non-*g* factors have negligible predictive power. In addition, the results are consistent with investment theories. SAT math residuals presumably reflect investment in math, which boosts STEM GPAs but retards humanities GPAs. In contrast, SAT verbal residuals presumably reflect investment in verbal areas, which yields the opposite pattern of effects.

## 5. Ability Tilt Predicts Diverse Criteria

Another non-*g* factor with predictive power is ability tilt, defined as the within-subject difference in math and verbal scores on standardized tests such as the SAT and ACT. The within-subject difference yields two types of tilt: *math tilt*, which occurs when math scores are higher than verbal scores, and *verbal tilt*, which occurs when verbal scores are higher than math scores. Both types of tilt are unrelated to *g* but, like the SAT and ACT non-*g* residuals, still predict STEM and humanities criteria.

Lubinski, Benbow, and colleagues (for a review see, [18]; see also, [19,20,21,22]) were the first to define and systematically examine ability tilt in the Study of Mathematically Precocious Youth (SMPY). The SMPY is a longitudinal study of intellectually gifted youth (top 1% or higher) who took the SAT around age 12 years and were tracked into adulthood. The SMPY estimated *ability level* using SAT sum scores (math plus verbal), which correlate strongly with *g*, and *ability tilt* using SAT difference scores (math minus verbal), which are unrelated to *g*. Whereas ability level correlated positively with adult achievements (e.g., income and education), ability tilt (math or verbal) predicted the domain of achievement. Math tilt predicted STEM achievements (STEM degrees, patents, engineering jobs), whereas verbal tilt predicted humanities achievements (e.g., humanities degrees, books published, journalism jobs) [18].

Would the results of the SMPY replicate with a representative sample? The question is important because the SMPY involves gifted subjects (top 1% in ability). Moreover, ability tilt is a type of ability specialization (math or verbal), which may vary with ability level. In particular, differentiation theories assume that cognitive abilities become more differentiated (and less *g* loaded) at higher ability levels, which are associated with more ability specialization (e.g., [23]). An implication is that ability specialization should be more pronounced for SMPY subjects than for a representative sample of (lower ability) subjects, who should show less ability specialization and less tilt, which is a type of ability specialization.

Coyle, Purcell, Snyder, and Richmond ([24]; see also, [25]) examined ability tilt using a representative sample with a wider range of ability. The sample was drawn from the NLSY, a representative sample of youth in the United States. (The NLSY was also used in the studies of non-*g* residuals.) As in the studies of non-*g* residuals (e.g., [15]), the ASVAB estimated two academic abilities (math, verbal) and two non-academic abilities (speed, shop). Ability tilt (math tilt and verbal tilt) was based on math and verbal scores from the SAT and ACT, which are typically taken in grades 11 or 12, and from the PSAT, which is typically taken in grade 10. Tilt scores on the SAT, ACT, and PSAT were correlated with the four ASVAB abilities (after removing *g*) and also with college majors and jobs in STEM (e.g., engineering) and humanities (e.g., English).

Coyle et al.’s ([24]; see also, [25]) results confirmed the results of the SMPY (cf. [18]). Math tilt on all three tests (SAT, ACT, PSAT) correlated positively with ASVAB math ability and negatively with ASVAB verbal ability, whereas verbal tilt showed the opposite pattern (|*M*_β_| ≈ 0.28). (Math and verbal tilt correlated negligibly with the non-academic shop and speed abilities, demonstrating divergent validity.) In addition, math tilt predicted STEM majors and jobs, whereas verbal tilt predicted humanities majors and jobs (|*M*_β_| ≈ 0.35). The results confirm the predictive power of non-*g* factors and are inconsistent with the primacy of *g* hypothesis, which assumes that non-*g* factors have negligible predictive validity. In addition, the results are consistent with investment theories ([16], pp. 138–146). Ability tilt presumably reflects investment in math or verbal abilities, which boost similar abilities and preferences (e.g., math tilt and STEM) and inhibit competing abilities and preferences (e.g., math tilt and humanities).

Coyle et al.’s [24] results were extended in separate analyses of sex differences [25] and race differences (whites and blacks) [26]. The results indicated that mean levels of math tilt were higher for males (than females) and for whites (than blacks), whereas mean levels of verbal tilt were similar between groups. Similar to Coyle et al.’s [24] initial research (with undifferentiated groups), tilt was correlated with ASVAB abilities, college majors, and jobs, separately for each sex (males and females) and race (whites and blacks). The results replicated for all groups. Despite group differences in mean levels of tilt, math tilt generally predicted STEM criteria (STEM jobs, majors, abilities), whereas verbal tilt generally predicted humanities criteria (humanities jobs, majors, abilities). The results suggest that tilt relations (with diverse criteria) are not specific to a particular sex or race but apply to all groups.

### A Non-g Nexus Involving Non-g Group Factor Residuals

**Figure 4:**
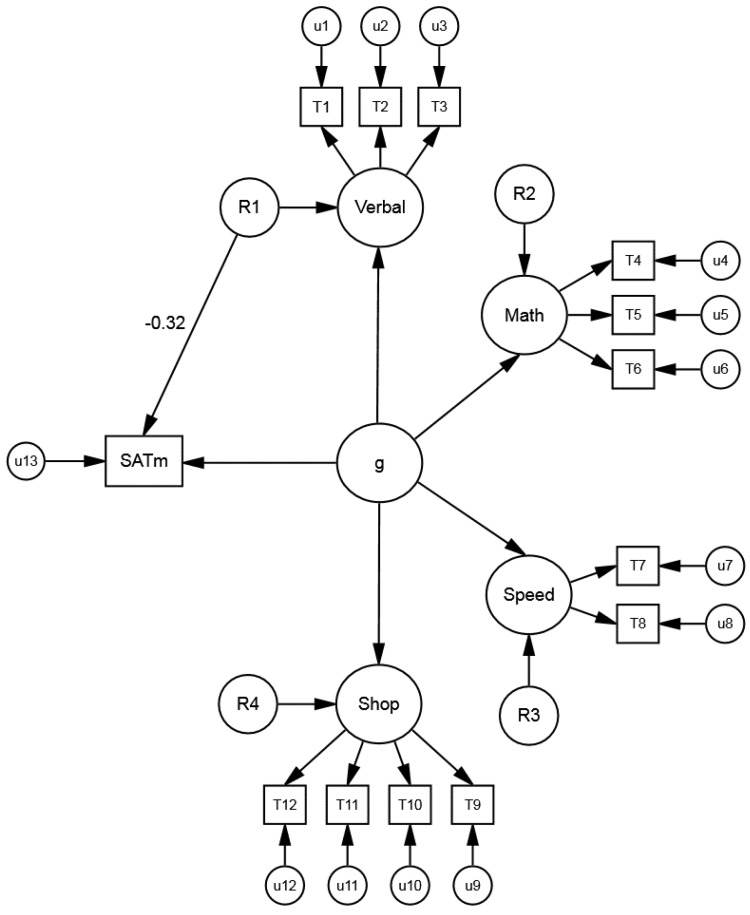
Model of *g* with ASVAB abilities (math, verbal, speed, shop). The symbol “R1” represents the ASVAB verbal non-*g* residuals, obtained after removing *g*. The R1→SAT math path estimates the relation of the ASVAB verbal non-*g* residuals with the SAT math subtest (β = −0.32). Figure adapted from Coyle [27].

Whereas the prior studies focused on non-*g* factors of a single test (e.g., SAT or ACT), a recent study by Coyle [27] focused on non-*g* residuals of group factors (based on multiple tests). The group factors were based on the ASVAB abilities (math, verbal, shop, speed) and were estimated using multiple tests with data from the NLSY (Figure 4). In general, group factors should yield more accurate estimates of non-*g* effects than individual tests (e.g., SAT and ACT), which are loaded with unique test-specific variance. As in the prior studies, the non-*g* residuals of the group factors were correlated with performance criteria (test scores and tilt scores on the SAT, ACT, and PSAT) and preference criteria (majors and jobs) in STEM and humanities.

Coyle’s [27] results confirmed the predictive power of non-*g* residuals of the ASVAB group factors. Math residuals correlated positively with math/STEM criteria (test scores, tilt scores, college majors, jobs) and negatively with verbal/humanities criteria. In contrast, verbal residuals showed the opposite pattern. The mean effect size was medium to large (|*M*_β_| = 0.51) [14]. (The shop and speed residuals generally correlated negligibly with all criteria, providing divergent validity.) The results were interpreted in terms of a non-*g* nexus involving non-*g* residuals of group factors and diverse criteria. The non-*g* nexus complements Jensen’s ([7], pp. 544–583) notion of a “*g* nexus” involving *g* and diverse criteria. Like the tilt effects, the non-*g* nexus suggests trade-offs, with investment in a specific ability (reflected by non-*g* residuals) boosting similar abilities (e.g., math) but inhibiting competing abilities (e.g., verbal).

## 6. Standing on the Shoulders of Giants: Other Research on Non-*g* Factors

Isaac Newton ([28], p. 416) said, “If I have seen further it is by standing on ye sholders of Giants”. In this section, I would like to acknowledge some key studies that inspired my research on non-*g* factors and that bolster the predictive power of non-*g* factors. The studies examine non-*g* factors for countries other than the United States, cognitive abilities other than those sampled by the ASVAB, SAT, and ACT, and ability levels other than those sampled by the NLSY.

Calvin, Fernandez, Smith, Visscher, and Deary [29] examined non-*g* residuals linked to specific abilities (math and verbal) in 175,000 English students (in the UK) who received the Cognitive Abilities Test (CAT), which includes tests of verbal, quantitative, and non-verbal reasoning. Non-*g* residuals of each test were estimated (after removing *g*), and correlated with each other and with the raw scores of each test. Consistent with Coyle et al.’s [15] results, the math residuals correlated positively with the math (raw) scores and negatively with the verbal scores, whereas the verbal residuals showed the opposite pattern. The effect sizes ranged from moderate to strong (|*M_r_*| = 0.31, range = −0.21 to 0.40) ([29], p. 427). Moreover, the effects were based on a large and representative sample of participants and tests, inspiring confidence in the results.

Johnson and Bouchard [30] analyzed data from the Minnesota Study of Twins Reared Apart (MISTRA) (*N* = 436) using the Verbal-Perceptual-Rotation (VPR) model. The VPR model involves a fourth-stratum *g*, three broad third-stratum factors (verbal, perceptual, rotation), and several narrow second-stratum factors linked to specific test performance (e.g., verbal, scholastic, number, speed, spatial, image rotation). The non-*g* residuals of the second-stratum factors (obtained after removing *g*) were correlated with each other ([30], p. 31). A key finding was the strong negative correlations of the verbal residuals with the spatial and rotational residuals (*M_r_* = −0.55), which predict math/STEM criteria (e.g., [25,31]). The residual correlations of the VPR verbal and spatial abilities are analogous to the residual correlations of the ASVAB verbal and math abilities. Both sets of correlations are negative, which suggests a tradeoff between competing abilities (e.g., verbal-spatial or verbal-math). The tradeoff is consistent with investment theories, which predict that investment in one ability (e.g., verbal) comes at the expense of investment in competing abilities (e.g., spatial), yielding negative effects.

As discussed above, Lubinski, Benbow, and colleagues published seminal research on ability tilt using SAT scores from gifted students (top 1% in ability) in the SMPY (for a review, see [18]). SAT tilt scores (math minus verbal) were unrelated to SAT sum scores (math plus verbal), which correlate strongly with *g* (e.g., [2]). Despite being unrelated to *g*, tilt scores predicted diverse criteria in STEM and humanities. The criteria included favorite course in high school, college major, graduate degrees, technology patents, books published, and occupations. In general, math tilt predicted STEM criteria, whereas verbal tilt predicted humanities criteria. The results laid a foundation for my studies on tilt and non-*g* residuals using a representative sample from the NLSY (e.g., [27]).

Together, the studies reviewed in this section, along with my studies, confirm the predictive power of non-*g* factors (ability tilt and non-*g* residuals) for diverse criteria (e.g., GPAs, college majors, college degrees, jobs). Collectively, the studies yield a pattern of results that replicates with different samples (NLSY, SMPY, MISTRA), tests (SAT, ACT, PSAT, ASVAB, CAT), abilities (math, verbal, spatial), and models (VPR model, ASVAB model), supporting the robustness of non-*g* effects.

## 7. Future Directions: There is Nothing More Practical than a Good Theory

Kurt Lewin ([32], p. 169) said, “There is nothing more practical than a good theory”. Good theories generate new hypotheses, facilitate interpretation of results, and guide future research. This last section reviews areas for future research, focusing on theories related to non-*g* factors. The theories include investment theories, Spearman’s Law of Diminishing Returns (SLODR), and the Cognitive Differentiation-Integration Effort (CD-IE) model. The section also discusses alternative types of ability tilt (e.g., technical tilt) and alternative non-*g* factors (e.g., non-academic factors) and concludes with a magnification model of non-*g* factors.

As noted, investment theories are widely used to interpret non-*g* effects ([16], pp. 138–146; see also, [25,26,27]). Such theories assume that differential investment of time and effort influences specific abilities (unrelated to *g*) and preferences. Investment in STEM is assumed to boost math abilities, which leads to math tilt and STEM preferences. In contrast, investment in the humanities is assumed to boost verbal abilities, which leads to verbal tilt and humanities preferences. Future research should examine whether continued investment (over time) in a particular area influences non-*g* effects. One prediction is that continued investment would boost specific abilities and strengthen non-*g* effects. Such a pattern may be observed in university settings, with continued investment in a particular field of study (e.g., math/STEM or verbal/humanities) increasing the influence of non-*g* effects (e.g., ability tilt and non-*g* residuals).

Another relevant theory is Spearman’s Law of Diminishing Returns (SLODR). SLODR is based on Spearman’s ([33], p. 219) observation that correlations among mental tests generally decrease at higher ability levels, presumably because tests become less loaded with *g* (variance common to tests) and more loaded with non-*g* factors (variance unrelated to *g*). SLODR has received empirical support. In general, correlations and *g* loadings of tests decrease, and non-*g* effects increase, at higher ability levels [34]. The decrease in *g* (and increase in non-*g* effects) is assumed to reflect cognitive differentiation and specialization at higher ability levels, which boosts specialized abilities. The specialized abilities include verbal and math abilities (e.g., tilt and non-*g* residuals), which are unrelated to *g*. Future research should examine whether the effects of tilt and non-*g* residuals increase at higher ability levels, as predicted by SLODR.^3^

A third theory is based on the Cognitive Differentiation-Integration Effort (CD-IE) model [35,36]. CD-IE is an evolutionary model with implications for investment in mating effort versus ability specialization in specific areas (e.g., math or verbal). CD-IE distinguishes between fast and slow life histories, which are associated with different levels of mating effort versus educational specialization, which increases ability specialization (and non-*g* effects). Fast life histories are associated with high levels of mating effort and less educational investment, yielding less ability specialization and weaker non-*g* effects. In contrast, slow life histories are associated with low levels of mating effort and more educational investment, yielding more ability specialization and stronger non-*g* effects. The predictions of the CD-IE model have been confirmed using ASVAB scores from the NLSY (1979 cohort), which showed increased non-*g* variance (reflecting specialization) at slower life history levels [36]. Future research should examine whether life history influences ability tilt, non-*g* residuals, and other non-*g* factors. Based on CD-IE theory, non-*g* factors should become more pronounced at slower life history speeds, reflecting greater educational specialization and less investment in mating effort.

It should be noted that all three theories (investment theories, SLODR, CD-IE) predict that non-*g* effects increase nonlinearly with ability specialization (cf. [1,27]). In particular, non-*g* effects are expected to strengthen over time with factors that influence ability specialization (e.g., ability level, life history, education level), which magnify non-*g* effects. The predicted pattern is consistent with niche picking theories [37] and experience producing drive theories [38]. Both theories assume that non-*g* effects are magnified over time as people seek out and select activities compatible with their predispositions. The predispositions include preferences for specific activities (e.g., STEM or humanities), which accelerate the development of specific abilities and magnify non-*g* effects.

Another area for future research concerns alternative types of ability tilt. Tilt is typically based on the difference between math and verbal scores on standardized tests (e.g., SAT, ACT). The difference yields math tilt (math > verbal) and verbal tilt (verbal > math). Future research could explore two other types of tilt: *spatial tilt*, defined as the difference between spatial scores and other scores (e.g., math or verbal), and *technical tilt*, defined as the difference between shop/technical scores and other scores (e.g., math or verbal). Spatial tilt would reflect elevated spatial abilities, which predict STEM achievements [31]. Technical tilt would reflect elevated technical abilities (e.g., cars, electronics, tools), which may predict non-academic pursuits and jobs (e.g., mechanic, carpenter). Both types of tilt could be measured using tests of spatial and technical abilities (e.g., the ASVAB). In addition, both types of tilt could be used to examine predictions related to ability specialization. As with other types of tilt, high levels of spatial and technical tilt would be predicted at higher ability levels and at slower life histories, which accelerate ability specialization. In contrast, lower levels of spatial and technical tilt would be predicted at lower ability levels and at faster life histories, which inhibit specialization.

A final suggestion, related to the prior one (on tilt measures), concerns the abilities sampled in non-*g* studies, which focus on academic abilities (math and verbal). An open question is whether similar results would be found for non-academic abilities such as shop or technical abilities. Preliminary evidence on the question comes from Coyle’s ([27], p. 22) analysis of non-*g* residuals for the non-academic shop factor (based on the ASVAB), which was correlated with math and verbal test scores (on the SAT and ACT). The results indicated significant (but weak) relations between the non-*g* residuals of the shop factor and the math and verbal test scores (*M*_β_ ≈ −0.12), indicating that strong non-academic abilities were associated with weak academic abilities. The results suggest a tradeoff in investment in non-academic abilities (shop) and academic abilities (math and verbal), yielding negative effects. Further research is needed to substantiate non-*g* effects with other non-academic abilities (e.g., technical tilt) and to examine whether the effects vary with ability specialization factors (e.g., life history and ability level). In addition, future research could examine other non-academic traits such as social intelligence and Big Five personality traits. Possible candidates include emotional intelligence, agreeableness, and theory of mind, which may predict economic and social criteria (e.g., wealth, trust, prosocial norms) beyond *g* [39].

**Figure 5:**
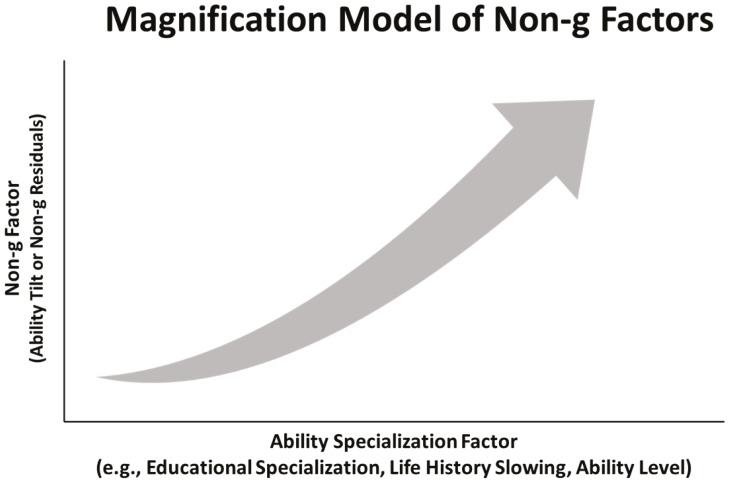
Magnification model of non-*g* factors. Non-*g* effects are predicted to strengthen nonlinearly with ability specialization factors (e.g., ability level, life history, education).

A *magnification model* summarizes the predictions related to ability specialization and non-*g* factors (Figure 5). The model predicts that non-*g* effects are magnified with increases in ability specialization factors (e.g., life history slowing, educational specialization, ability level). The predictions are depicted in Figure 5, which plots a nonlinear relationship between a non-*g* factor (e.g., ability tilt) and an ability specialization factor. Non-*g* factors (*y*-axis) include ability tilt and non-*g* residuals. Non-*g* effects are assumed to strengthen nonlinearly with ability specialization factors (*x*-axis). The expected increase in non-*g* effects can be formally tested by regressing a non-*g* factor (e.g., tilt level) on the linear and quadratic terms of a specialization factor. A key prediction is that a significant (and positive) quadratic term should account for additional variance beyond the linear term, indicating that non-*g* effects increase nonlinearly as a function of the ability specialization factor.^4^

## 8. Conclusions

The research reviewed here demonstrates the predictive power of non-*g* factors (e.g., ability tilt and non-*g* residuals). In general, non-*g* factors correlate positively with complementary criteria (e.g., math tilt and STEM criteria) and negatively with non-complementary criteria (e.g., math tilt and humanities criteria). The results are consistent with investment theories, which assume that investment in specific abilities (e.g., math/STEM) enhances complementary abilities and inhibits competing abilities (e.g., verbal/humanities). Future research should examine whether non-*g* effects increase with continued investment and ability specialization factors (e.g., life history slowing, ability level, educational specialization).

## Footnotes

1 Peterson and Brown ([12], p. 180) show that the relation between β and *r* is independent of sample size and number of predictors and that the imputation of *r* (given β) yields an estimate similar to the population statistic (ρ) (for a criticism of Peterson and Brown’s [12] approach, see [13]). Given the robust relationship between β and *r*, βs of 0.10, 0.30, and 0.50 could be described as small, medium, and large, respectively, using Cohen’s [14] criteria for correlations.

2 The main analyses analyzed SAT and ACT composite scores, which were the sum of the math and verbal subtest scores. The results replicated in separate analyses of SAT and ACT subtest scores.

3 Preliminary support for SLODR comes from Coyle’s [26] study of tilt effects for whites and blacks, two groups that show an average differences in *g* (favoring whites) of about 1 *SD*. In general, tilt levels were higher, and tilt relations with specific abilities were stronger, for whites than for blacks (e.g., [26], p. 32). Such a pattern is consistent with SLODR, which assumes that non-*g* effects (e.g., tilt effects) should be stronger for higher ability groups than for lower ability groups.

4 The predictions of the magnification model should be tested after correcting for measurement error, which can increase the predictive power of *g* relative to non-*g* factors ([40]; see also, [41]). In addition, corrections for shrinkage should be used to avoid capitalization on chance (e.g., [42], p. 515; see also, [43]), and corrections for range restriction should be used to avoid variance compression, which can reduce effects sizes.
